# *In vitro* and *in vivo* efficacy of combinations of colistin and different endolysins against clinical strains of multi-drug resistant pathogens

**DOI:** 10.1038/s41598-020-64145-7

**Published:** 2020-04-28

**Authors:** Lucia Blasco, Anton Ambroa, Rocio Trastoy, Ines Bleriot, Miriam Moscoso, Laura Fernández-Garcia, Elena Perez-Nadales, Felipe Fernández-Cuenca, Julian Torre-Cisneros, Jesus Oteo-Iglesias, Antonio Oliver, Rafael Canton, Tim Kidd, Ferran Navarro, Elisenda Miró, Alvaro Pascual, German Bou, Luis Martínez-Martínez, Maria Tomas

**Affiliations:** 1 Microbiology Department-Research Institute Biomedical A Coruña (INIBIC), Hospital A Coruña (CHUAC), University of A Coruña (UDC), A Coruña, Spain; 2 Unit of Microbiology, University Hospital Reina Sofía, Department of Microbiology, University of Córdoba, Maimonides Biomedical Research Institute of Cordoba (IMIBIC), Cordoba, Spain; 3 Clinical Unit for Infectious Diseases, Microbiology and Preventive Medicine, Hospital Universitario Virgen Macarena / Department of Microbiology and Medicine, University of Seville/ Biomedicine Institute of Seville (IBIS), Seville, Spain; 4 Reference and Research Laboratory for Antibiotic Resistance and Health Care Infections, National Centre for Microbiology, Institute of Health Carlos III, Majadahonda, Madrid, Spain; 5 Microbiology Department-Research Institute Biomedical Islas Baleares (IdISBa), Hospital Son Espases, Palma de Mallorca, Spain; 6 Microbiology Department-Research Institute Biomedical Ramón and Cajal (IRYCIS), Hospital Ramón and Cajal, Madrid, Spain; 7 School of Chemistry and Molecular Biosciences and Child Health Research Centre, The University of Queensland, Brisbane, QLD Australia; 8 Microbiology Department-Sant Pau Hospital, Barcelona, Spain; 9 Study Group on Mechanisms of Action and Resistance to Antimicrobials (GEMARA) of the Spanish Society of Infectious Diseases and Clinical Microbiology (SEIMC), Madrid, Spain; 10 Spanish Network for Research in Infectious Diseases (REIPI), Seville, Spain

**Keywords:** Drug discovery, Microbiology

## Abstract

The emergence of multidrug resistant (MDR) pathogenic bacteria is jeopardizing the value of antimicrobials, which had previously changed the course of medical science. In this study, we identified endolysins ElyA1 and ElyA2 (GH108-PG3 family), present in the genome of bacteriophages Ab1051Φ and Ab1052Φ, respectively. The muralytic activity of these endolysins against MDR clinical isolates (*Acinetobacter baumannii*, *Pseudomonas aeruginosa* and *Klebsiella pneumoniae*) was tested using the turbidity reduction assay. Minimal inhibitory concentrations (MICs) of endolysin, colistin and a combination of endolysin and colistin were determined, and the antimicrobial activity of each treatment was confirmed by time kill curves. Endolysin ElyA1 displayed activity against all 25 strains of *A. baumannii* and *P. aeruginosa* tested and against 13 out of 17 strains of *K. pneumoniae*. Endolysin ElyA2 did not display any such activity. The combined antimicrobial activity of colistin and ElyA1 yielded a reduction in the colistin MIC for all strains studied, except *K. pneumoniae*. These results were confirmed *in vivo* in *G. mellonella* survival assays and in murine skin and lung infection models. In conclusion, combining colistin (1/4 MIC) with the new endolysin ElyA1 (350 µg) enhanced the bactericidal activity of colistin in both *in vitro* and *in vivo* studies. This will potentially enable reduction of the dose of colistin used in clinical practice.

## Introduction

The worldwide emergence of multidrug resistant (MDR) microorganisms that are refractory to treatment with current therapeutic agents has highlighted the urgent need for new classes of antimicrobial agents^1^. The World Health Organization (WHO) has recently published a list of “priority pathogens” which includes those microorganisms that are considered a serious threat to human health and for which new anti-infective treatments are urgently needed. The list includes carbapenem-resistant *A. baumannii*, *P. aeruginosa* and *K. pneumoniae* clinical isolates^2^.

One consequence of the emergence of the MDR bacteria is a return to the use of previously abandoned antimicrobials. This is the case with colistin (polymyxin E), a cationic peptide which disturbs the stability and increases the permeability of the outer membrane via electrostatic interactions and cationic displacement of the lipopolysaccharide. Although colistin exerts antimicrobial effects, it also has nephrotoxic effects and has gradually been abandoned and substituted by other, better-tolerated antibiotics^3,4^. Combining new antimicrobial agents with old antibiotics such as colistin is a new strategy in the development of novel treatments against MDR microorganisms.

In recent years, a novel drug discovery approach has explored endolysin enzymes (also referred to as enzybiotics), which are encoded by bacteriophages (viruses which infect bacteria) (5). Endolysins are actively produced during the lytic cycle and exert antibacterial activity by degrading peptidoglycan in the bacterial cell wall^5,6^.

Endolysins are highly evolved enzymes produced by bacteriophages to digest the bacterial cell wall at the end of their replication cycle and release the phage progeny. Endolysins target the integrity of the cell wall and attack one of the major bonds in the peptidoglycan layer. They can be classified into five groups according to the cleavage site: N-acetyl-β-D-muramidase (lysozymes); N-acetyl-β-D-glucosaminidases (glycosidases); lytic transglycosylase; N-acetylmuramoyl-L-alanine amidases and L-alanoyl-D-glutamate endopeptidases^7,8^.

Endolysins are good candidates as new antimicrobial agents against Gram-positive bacteria, in which the peptidoglycan layer of the cell wall is exposed to the medium. Several studies have evaluated the potential use of endolysins against Gram-positive bacteria such as *Staphylococcus aureus, Streptococcus agalactiae*, *Streptococcus pneumoniae* and *Streptococcus pyogenes* in animal models of human infections and diseases^9–16^. In Gram-negative bacteria, the outer membrane acts as a barrier to many endolysins, and very few endolysins with exogenous activity against Gram-negative bacteria have been described (many are biotechnologically engineered)^17–20^. Endolysins can attack Gram-negative bacteria when the outer membrane is previously permeabilized with agents such as EDTA, which destabilizes the lipopolysaccharides of the outer membrane; however, the combination of endolysin and EDTA is limited to topical treatment of localized infections^21,22^. In the search for alternative methods of killing MDR bacteria such as *A. baumannii*, *P. aeruginosa* and *K. pneumoniae*, various researchers have considered increasing the muralytic activity of endolysins by combining them with different antibiotics to take advantage of synergistic responses^22,23^.

In this study, we identified and characterized an endolysin, named ElyA1, isolated from the *A. baumannii* Ab105 (ROC0034a) bacteriophage Ab1051Φ. The endolysin displayed muralytic activity against a broad spectrum of MDR organisms. In addition, combining endolysin ElyA1 with colistin (polymyxin E) enhanced the susceptibility of the tested strains by at least four times (relative to the susceptibility to colistin alone), thus highlighting the potential of endolysin ElyA1 as a candidate antibacterial agent. This effect was confirmed by an *in vivo* test, in which the survival of the *G. mellonella* larvae increased when colistin (¼ MIC) was supplemented with endolysin ElyA1. Another endolysin from the same family, named ElyA2, was identified in the *A. baumannii* Ab105 bacteriophage Ab1052Φ, but did not display muralytic activity.

## Results

### Identification of endolysins ElyA1 and ElyA2

**Figure 1:**
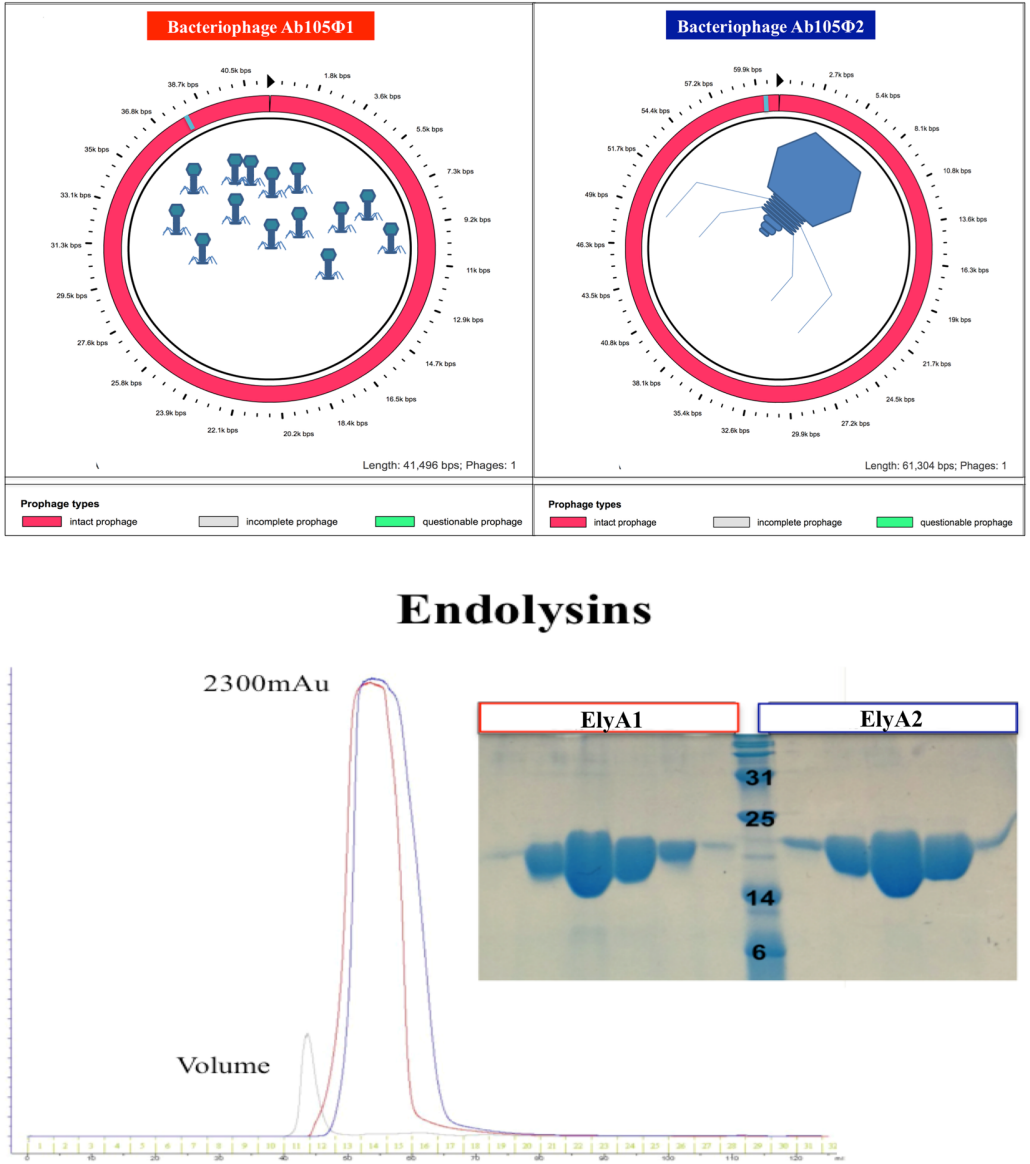
Genome of the bacteriophages Ab105Φ1 (GenBank: KT588074.1) and Ab105Φ2 (GenBank: KT588075.2) by figure modified with PHAST software (http://phast.wishartlab.com) (60). SDS-PAGE purification of the endolysins ElyA1 and ElyA2 (chromatographic study).

The 546 bp gene coding for endolysin ElyA1 was identified as an ORF (Open Reading Frame) encoding a protein of 181 aa (GenBank: ALJ99090.1) and molecular weight, 20.22 kDa (Fig. 1). The protein sequence was analysed with InterProScan and classified as a lysozyme (N-acetylmuramidase) with a C-terminal domain corresponding to the glycoside hydrolase superfamily 108 and also a peptidoglycan binding domain PG3 at the N-terminal end.

Protein homology analysis revealed a high level of homology (>80%) with a group of 9 endolysins from *A. baumannii* bacteriophages belonging to the same protein family as ElyA1^20^.

The 543 bp gene coding for endolysin ElyA2 was identified as an ORF encoding a protein of 180 aa (GenBank: ALJ99174.1) and molecular weight 20.19 kDa (Fig. 1). The sequence analysis revealed that the ElyA2 protein is also a lysozyme (N-acetylmuramidase), with a C-terminal domain corresponding to the glycoside hydrolase superfamily 108, and also a peptidoglycan binding domain PG3 at the N-terminal end.

Like the ElyA1 protein, this enzyme displays a high degree of homology (>80%) with the same group of 9 endolysins and also 90% homology with the ElyA1 protein^20^.

### Characterization of endolysin muralytic activity

**Figure 2:**
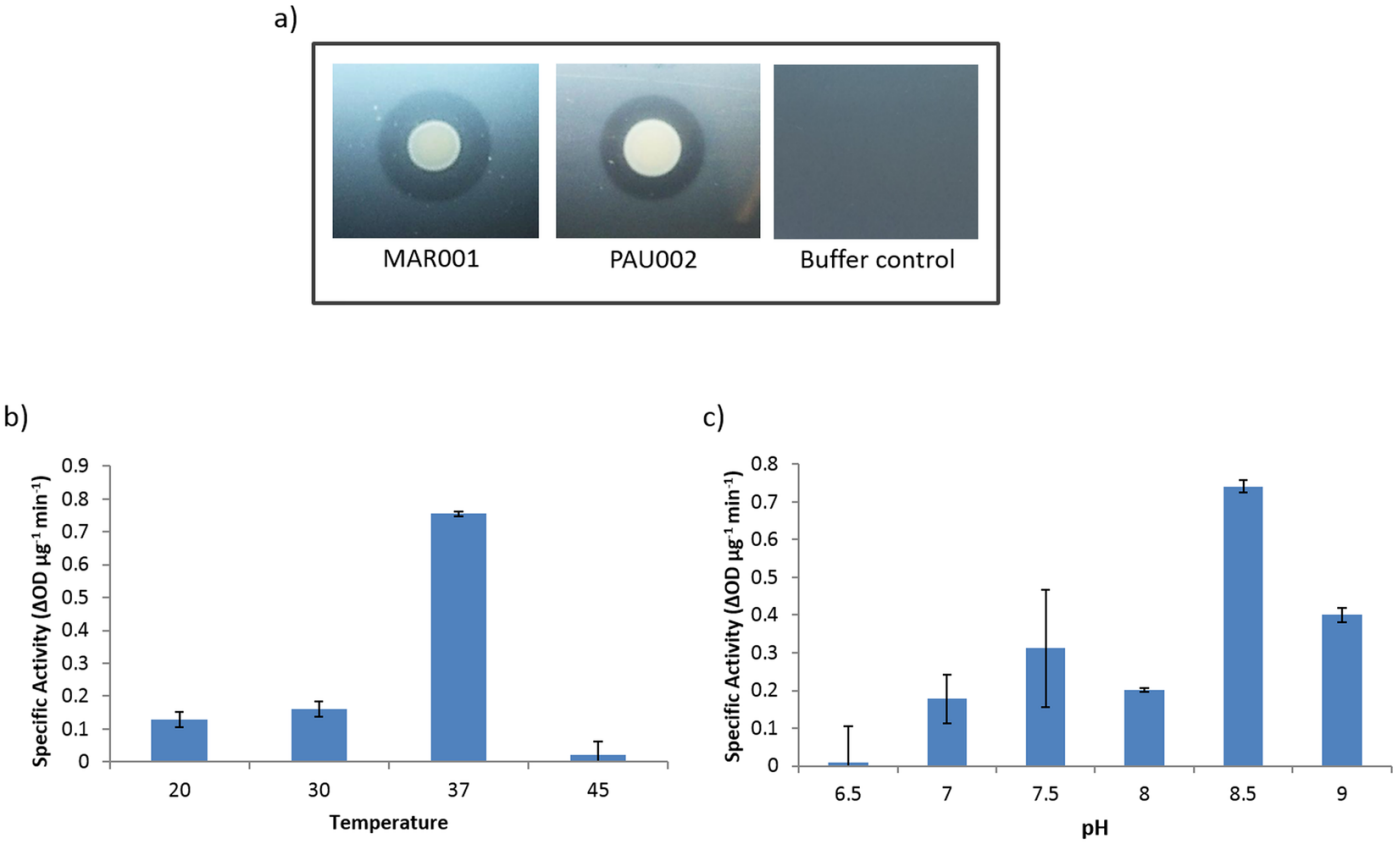
Characterization of enzymatic activity: (**a**) Muralytic activity of ElyA1 was determined by spotting ElyA1 and endolysin buffer as a negative control in an overlay of two Gram-negative *Acinetobacter baumannii* clinical isolates, MAR001 and PAU002; (**b**) pH range and (**c**) temperature range were determined by the specific activity, measured as the difference in optical density of the culture per µg of enzyme and minute.

In the initial screening of the muralytic activity of the purified endolysin ElyA1 in the overlay plates with Gram-negative bacteria, a halo appeared around the lysis zones for both strains of *A. baumannii* tested (Fig. 2a).

The muralytic activity of this enzyme was characterized using the Gram-negative bacteria *A. baumannii* Ab105 as substrate, as this is the host strain for the phage Ab105Φ1. The enzymatic activity was measured after incubation at different temperatures and pH. The maximum activity was obtained after incubation for 10 min at pH8.5 and 37 °C (Fig. 2b,c). In addition, the muralytic activity on the Ab105 cells was assayed directly or after treatment of the cells with EDTA to permeabilize the outer membrane. However, no activity was detected when the enzyme was added directly to the cells whose outer membrane had not been permeabilized with EDTA, and in this case the cells also tended to aggregate (data not shown).

**Figure 3:**
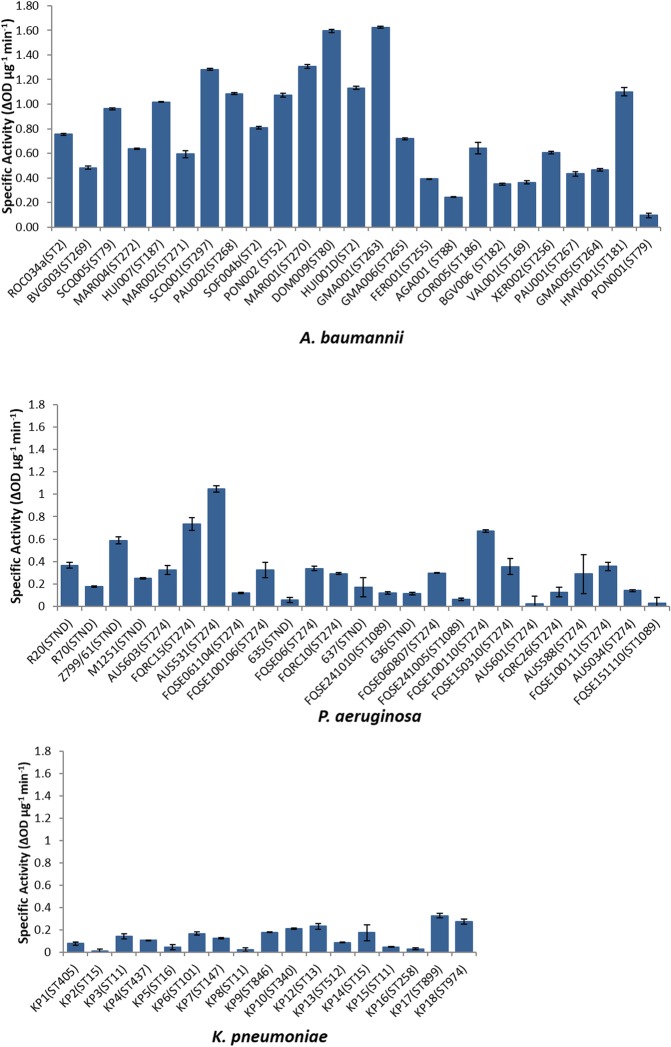
Specific activity of endolysin ElyA1 tested in clinical isolates from different mutlilocus sequence types (STs) of three Gram-negative members of the ESKAPE group: *Acinetobacter baumannii, Pseudomonas aeruginosa and Klebsiella pneumoniae*.

The antibacterial assays showed a broad lytic spectrum of activity against the strains of the three species tested (Fig. 3). As expected because of the origin of the *A. baumannii* endolysin, the activity was highest among the 25 *A. baumannii* strains tested. Although the activity was more variable in *P. aeruginosa*, muralytic activity against all of the strains was detected. Finally, endolysin ElyA1 was active against 13 of the 17 *K. pneumoniae* strains, although at lower levels than in *A. baumannii* and *P. aeruginosa*. The strains of the three species tested belonged to different strain types (STs), but the susceptibility to endolysin ElyA1 was not correlated with the ST.

Tests of the muralytic activity of endolysin ElyA2 did not detect any activity under any the conditions assayed. On the contrary, this enzyme induced aggregation of the cells at all the enzyme concentrations tested, both in the cells treated previously with EDTA and in those with an intact outer membrane.

### **Combined activity of endolysin ElyA1 and colistin in*in vitro*assays**

**Figure 4:**
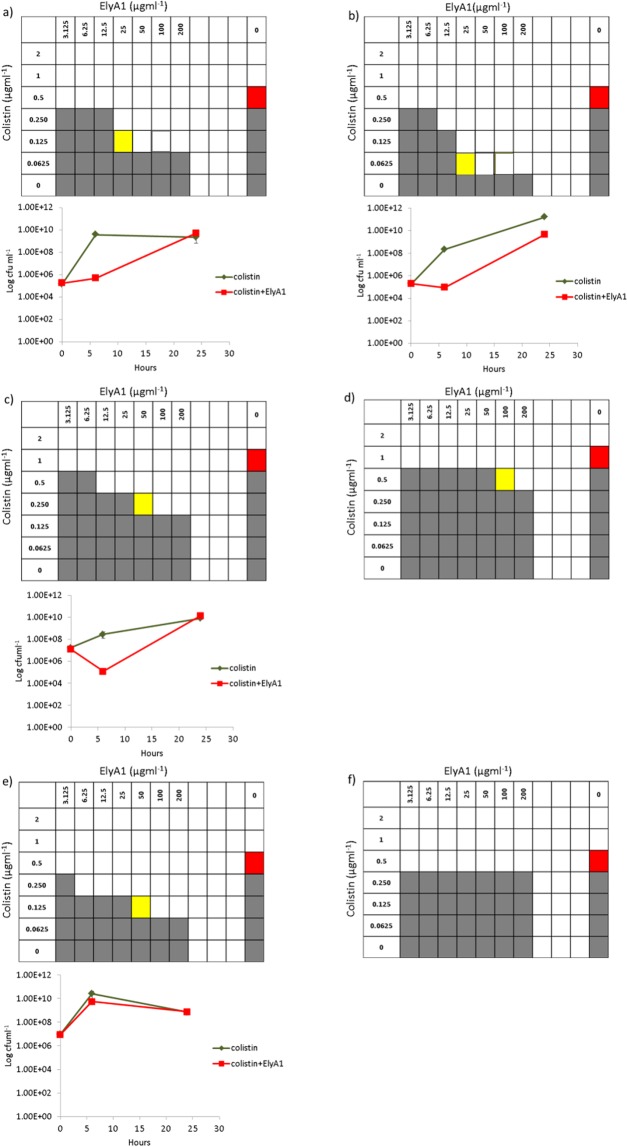
*In vitro* bactericidal activity of colistin in combination with endolysin ElyA1 measured by MIC and time kill curves in *Acinetobacter baumannii* strains GMA001 (**a**) and PON001 (**b**); *Pseudomonas aeruginosa* strains AUS531 (**c**) and AUS601 (**d**); *Klebsiella pneumoniae* strains KP17 (**e**) and KP16 (**f**). The time kill curves were only constructed for strains in which there was a fourfold reduction in colistin MICs (red square) when used in combination with endolysin ElyA1 (yellow square).

As ElyA1 is only active when the outer membrane of the target bacterial cell is solubilized, the MIC of the endolysin could not be determined using the microdilution checkerboard test. We therefore aimed to detect any decrease in the colistin MICs when used in combination with endolysin ElyA1. The addition of endolysin ElyA1 yielded a fourfold reduction in the colistin MICs in four of the six strains tested (*A. baumannii* GMA001 and PON001, *P. aeruginosa* AUS531 and *K. pneumoniae* KP17) (Fig. 4). By contrast, only a twofold reduction in the colistin MIC was observed with *P. aeruginosa* AUS601 and no decrease with *K. pneumoniae* KP16. The latter was consistent with the lack of enzymatic activity observed in the antibacterial assays (Fig. 4). Finally, no antimicrobial activity was detected when the combination was tested in the colistin resistant isolates (data not shown).

The results of the time kill curve assay confirmed the results of the microdilution checkerboard test (Fig. 4). A 2 log reduction in growth of both of the *A. baumannii* strains and *P. aeruginosa* AUS531 after 6 hours in the culture with 1/4 of colistin and endolysin ElyA1 was observed, indicating a synergetic reaction between colistin and endolysin ElyA1. By contrast, there was no reduction in growth in the *K. pneumoniae* KP17 culture.

**Activity of endolysin/colistin combinations in*in vivo assays*:**

**Figure 5:**
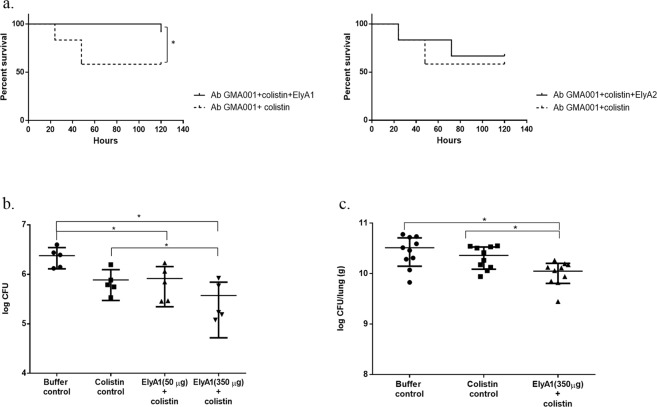
*In vivo* bactericidal activity of colistin in combination with endolysins ElyA1 and ElyA2. (**a**) Survival curves for *G. mellonella* larvae infected with *A. baumannii* clinical strain GMA001 and treated with colistin (1/4 MIC) and with colistin (1/4 MIC) combined with endolysin ElyA1 (25 μg/ml). Survival curves for *G. mellonella* larvae infected with *A. baumannii* clinical strain GMA001 and treated with colistin (1/4 MIC) or with colistin (1/4 MIC) combined with endolysin ElyA2 (25 μg/ml). This experiment was carried out with an appropriate survival control. *Statistically significant differences (*p* < 0.05) were determined by Graham-Breslow-Wilcoxon test (GraphPad Prism v.6); (**b**) Antimicrobial activity of endolysin ElyA1 in a murine skin model. CFU quantification in homogenized mouse skin after infection with *A. baumannii* GMA001 and treatment with colistin (1/4 MIC) in combination with different doses of endolysin ElyA1 (50 µg and 350 µg) or with buffer or colistin (controls). (**c**) Antimicrobial activity of ElyA1 in a murine lung infection model. CFU quantification in lungs after infection with *A. baumannii* GMA001 and treatment post-infection with colistin in combination with350 µg of ElyA1. * Statistically significant differences (*p* < 0.05) were determined by t-Student test (GraphPad Prism v.6).
  Larvae of the wax moth were infected with clinical strain *A. baumannii* GMA001. Survival of infected larvae treated with colistin (¼MIC) in combination with the ElyA1 (25 µg/ml) was significantly higher (*p* < 0.05) that that of larvae treated with colistin only (¼ MIC). Treatment with the combination of colistin (¼ MIC) and ElyA2 (25 µg/ml) did not yield significant differences (*p* > 0.05) relative to treatment with the colistin treatment, as ElyA2 did not display muralytic activity.
- Efficacy of ElyA 1 in the murine skin model (Fig. 5b)
  Mice superficial skin wounds were infected twice (on two consecutive days) with clinical strain *A. baumannii* GMA001. The wounds were treated with colistin in combination with different doses of ElyA1 (50 µg and 350 µg), a colistin control or a buffer control. The effectiveness of the treatments was established by counting the total number of CFUs in the skin wound. The cell counts were significantly lower (*p* ≤ 0.05; Student’s t-test) in the colistin combination treatments (with both doses of ElyA1) than in the buffer control. The cell counts in the 350 µg ElyA1 plus colistin treatment were also significantly lower (*p* ≤ 0.05; Student’s t-test) than in the colistin control.
- Efficacy of ElyA1 in treatment of lung infection (Fig. 5c)

- Mortality in the *in vivo Galleria mellonella* model (Fig. 5a)

Infected mouse lungs were only treated with the combination of colistin and 350 µg ElyA1, as colistin plus the lower dose of endolysin (50 µg) did not display any activity in the skin infection model.

Lung CFU counts were significantly lower (*p* ≤ 0.05; Student’s t-test) in the mice treated with the combination of colistin and ElyA1 than in the mice treated with buffer or with colistin. There were no significant differences in the CFU counts between the buffer control group and the colistin control group.

## Discussion

The discovery and development of novel antimicrobial agents to treat infections caused by the “priority” group of pathogens is a challenge facing the medical and research community^2^.

Enzybiotics have become the focus of attention of many research groups worldwide. Endolysins (one type of enzybiotics) are species or genus-specific enzymes that act by hydrolysing the peptidoglycan layer of the bacterial cell wall. There are no reports of bacteria developing resistance to endolysins, which is a problem in both antibiotic therapy and phage therapy^16^. Moreover, endolysins have been recognized in the US “National Action Plan for Combating Antibiotic-resistant Bacteria”^24^, which identified the use of “phage-derived lysins to kill specific bacteria while preserving the microbiota” as a key strategy to reduce the development of antimicrobial resistance due to the absence of toxicity in human cells^25,26^. Moreover, some endolysins have been found to display activity against sub-populations of microbes^27,28^ extracted from biofilm^29–31^ and to be useful in other innovative treatments.

The outer membrane of Gram-negative bacteria acts as a barrier preventing access of many endolysins to their natural target, the peptidoglycan layer. Different strategies have been used to address this problem, including solubilization of the outer membrane with EDTA, modification of the endolysin PGs by deletion or substitution, and the development of fusion proteins such as Artilysin-175 (Art-175). This protein is made by fusing the endolysin with a peptide, successfully enabling the enzyme to pass through the outer membrane^18,32,33^. Art-175 constituted by fusing antimicrobial peptide (AMP) sheep myeloid 29-amino acid peptide (SMAP-29) with endolysin KZ144 displayed muralytic activity in a *P. aeruginosa* isolate, and continuous exposure to Art-175 did not lead to the development of resistance^18^. By itself, SMAP-29 is cytotoxic to mammalian cells^34^; however, Art-175 exhibited little toxicity in L-292 mouse connective tissue.

As a new strategy, we combined the membrane-destabilizing effect of colistin, a cationic peptide used as an active outer membrane agent (but only as a “last-line” treatment due to concerns about its nephrotoxicity and neurotoxicity^35^), and two endolysins identified by our research group and belonging to a lysozyme-like family (GH108-PG3) never before used as antimicrobial treatment.

In this study, we identified two endolysins, ElyA1 and ElyA2, obtained from *A. baumannii* bacteriophage Ab1051Φ and Ab105Φ2, available in a collection of clinical strains of *A. baumannii* isolated during the II Spanish Multicentre Study GEIH/REIPI-*A.baumannii* 2000–2010 (Accession number PRJNA422585, Genbank Umbrella Bioproject)^36,37^.

Endolysins ElyA1 and ElyA2 are lysozyme-like proteins with a catalytic domain and a cell wall binding domain (CBD), responsible for recognition of the cell surface ligands and affinity for the bacterial substrate^6,38^. This structure is most commonly found in endolysins from bacteriophages that target Gram-positive bacteria. However, the PG_3 domain present in endolysins ElyA1 and ElyA2 has been identified in some Gram-negative bacteria and in a group of nine endolysins isolated from bacteriophages of *A. baumannii*; the domain shows high homology with ElyA1 and belongs to the same family (Fig. 1)^20,36,39^. The present findings regarding the molecular characteristics and comparative genomes in bacteriophage endolysins confirm previously reported findings^40^.

The bacteriophages from which these endolysins were isolated, Ab1051Φ and Ab105Φ2, occur in a large number of clinical isolates of *A. baummanii*^36^. The cell wall binding domain has been shown to be responsible for the specificity and affinity of the endolysins for its substrate^39^. However, endolysin ElyA1 displayed a broader spectrum of activity against strains of *A. baumannii* and many strains of *P. aeruginosa* belonging to the same order (*Pseudomonadales*), and to lesser extent against some strains of *K. pneumoniae* from another gammaproteobacterial order, *Enterobacterales*. In this case, the target of endolysin ElyA1, identified in peptidoglycan (PG) binding domains as a D-Asn^40^, is probably conserved among the *Pseudomonadales*, thus explaining the broad spectrum of action of this enzyme. Interestingly, we were not able to detect muralytic activity in endolysin ElyA2, because this enzyme induces aggregation of the cells *in vitro*. An aggregative effect was previously described in the endolysin phi12 isolated from a *S. aureus* bacteriophage, although the cause of the effect was unknown^41^. Autoaggregation has been suggested to occur in environmental stress caused by toxins, antibiotics, predation or low nutrients^42^.

In the present study, we used the cationic polymyxin antibiotic colistin to overcome the impenetrability of the outer membrane to endolysin ElyA1. Colistin disturbs the outer membrane via an electrostatic interaction with lipopolysaccharides and phospholipids present in the outer membrane^4^. The synergistic effect of colistin and endolysin LysABP-01 (a lysozyme-like protein from the GH19 family) on *A. baumannii* has previously been described^22^. Although the endolysin ElyA1 does not display exogenous activity, because of its inability to cross the outer membrane, this problem was largely overcome when the enzyme was used in combination with colistin. The antimicrobial activity of the combined therapy was higher than for both substances used alone, for all of the strains tested, except the *K. pneumoniae* strains. A reduction in the colistin MIC of at least fourfold was observed for all of the *A. baumannii* strains tested and for *P. aeruginosa* strain AUS531, and a corresponding twofold reduction was observed for *P. aeruginosa* strain AUS601. A reduction in the colistin MIC was also obtained for *K. pneumoniae* strain KP17, the strain most susceptible to endolysin ElyA1. The increased antimicrobial activity with endolysin ElyA1 and colistin was confirmed with an almost 3 log reduction in growth after 6 h in all strains tested, except *K. pneumoniae* KP17. Growth of the culture reached the same level as in the control after 24 h, probably due to degradation of the enzyme and colistin, as previously reported^22^. In all of the strains tested, the reduction in the colistin MIC was consistent with the muralytic activity of endolysin ElyA1 observed with those strains. No antimicrobial activity was observed when this assay was conducted with colistin-resistant strains, probably because of the inability of the enzyme to access the peptidoglycan layer, as the necessary destabilization of the outer membrane by the colistin was not produced in these isolates. However, several mechanisms of resistance to colistin have been described. In some mechanisms, the lipopolysaccharide is modified or is not produced, preventing binding of the colistin to the outer membrane. Other mechanisms include efflux pumps described in *A. baumannii* and inhibition of respiratory enzymes such as NADH oxidase in Gram-positive bacteria such as *Bacillus* spp. and NADH quinone oxidoreductase in *E. coli*. The activity of the enzyme is likely to be higher in the bacteria with colistin resistance mechanisms different from those involving modification of the lipopolysaccharides^43–49^. In Europe, the incidence of colistin resistant *A. baumannii* in intensive care units reached over 23% due to different mechanisms of resistance such as alterations in the lipopolysaccharide (LPS) as well as acquistion of *mcr* genes^50^. Because of the possible inability of these combinations to inhibit colistin resistant strains, further studies must be conducted with a range of different bacteria with different mechanisms of resistance to colistin with the aim of reducing the colistin MIC in combination with endolysin ElyA1.

The results obtained *in vitro* were confirmed with those of *in vivo* assays, as the survival of the infected *G. mellonella* larvae was higher when the worms were treated with a combination of a reduced (fourfold) MIC of colistin and endolysin ElyA1 than with colistin alone. As a control, the same assay was performed with endolysin ElyA2, in which no muralytic activity was detected, and there were no differences relative to treatment with colistin. As in *G. mellonella*, the antimicrobial activity of ElyA1 was confirmed *in vivo*. A combination of colistin and 350 µg of ElyA1 was used to treat the skin infection and lung infection in mice, yielding a significant reduction in the number of bacteria relative to treatment with colistin alone.

In conclusion, this is the first *in vitro* and *in vivo* study in which colistin has been combined with endolysin ElyA1 (glycosyde hydrolase superfamily 108) to treat infections caused by clinical MDR pathogens. This type of treatment may enable a reduction in the concentration of colistin used in antimicrobial treatments, thus also reducing the toxic side effects of the antibiotic. The broad spectrum of action of endolysin ElyA1 would enable the inclusion of more MDR Gram-negative bacteria as targets for the combined antimicrobial treatment.

## Materials and Methods

### Strains and culture conditions

The bacterial strains and plasmids used in this study included 25 *A. baumannii* MDR strains belonging to 22 different sequence types (STs) (Table 1). The strains were isolated from colonized or infected patients within the framework of the II Spanish Multicentre Study, in which 45 Spanish hospitals participated (GEIH-REIPI *Acinetobacter baumannii* 2000–2010, Genbank Umbrella Bioproject accession number PRJNA422585)^36,37^. The strains included 25 MDR clinical strains of *P. aeruginosa* (many included in CC274), all of which were isolated from cystic fibrosis patients, and 17 carbapenemase-producing strains of *K. pneumoniae*, which were isolated in 20 Spanish hospitals during the EuSCAPE project^51,52^. Moreover, *Escherichia coli* DH5α and Rosetta strains were used in cloning assays (Table 1).

**Table 1:** Description of the bacterial strains, plasmids and primers used in this study.

Strain, Plasmid, Primer, Strain	Description, Characteristics and Sequence	Origin and Reference
**Strain**		
*Acinetobacter baumannii*	25 clinical isolates (22 STs) from the II Spanish Multicentre Study (GEIH-*REIPI Acinetobacter baumannii* 2000–2010) (Accession number Genbank PRJNA422585)	27
*Pseudomonas aeruginosa*	25 clinical isolates (ST274 [n = 15]; ST1089 [n = 3]; ST not known [n = 7])	28
*Klebsiella pneumoniae*	17 clinical isolates belonging to 16 different STs	29
*Escherichia coli* DH5α	Strain using for cloning	Novagen
*Escherichia coli* Rosetta pLys-S	Strain for protein expression	Novagen
**Plasmid**		
pET-28a	Km^r,^, T7*lac*, His-Tag, T7-Tag, thrombine protease site	Novagen
**Primers**		
Forward	5′-AGTTCTGTTCCAGGGGCCCCATATGAACATTGAACAATATCTTGATGAA-3	This study
Reverse	5′-AGTGGTGGTGGTGGTGGTGCTCGAG**TCA**CATTGATACTCGATTAGCAAT-3′	This study

Abbreviations: ST; multilocus sequence type.

All strains were cultured in LB (Luria-Bertani) broth at 180 rpm and 37 °C. For solid medium, 2% of agar was added to LB broth. In the transformation assays, the medium was supplemented with 50 µg/ml of ampicillin.

### Identification and purification of endolysins ElyA1 and ElyA2

Endolysin gene prediction, from the genome of the bacteriophage Ab105Φ1 (GenBank: KT588074.1) and Ab105Φ2 (GenBank: KT588075.2)^36,53^ (Fig. 1), was performed with the bioinformatic tools PHASTER (Phage Search Tool Enhanced Release) and RAST (Rapid Annotation Using Subsystem). Protein homology analysis was performed by BLAST (Basic Local Alignment Search Tool), Clustal Omega and MView. Protein families were assigned using InterProScan, and the domain graphic was assigned with PROSITE MyDomains.

The endolysin genes were amplified by PCR from the genomic DNA of *A. baumannii* Ab105 (which contains the DNA of the prophages Ab105Φ1 and Ab105Φ2) and cloned into the expression vector pET-28a (Novagen). The recombinant plasmids were transformed into competent *E. coli* DH5α cells (Novagen) for DNA production and purification, and the integrity of both constructs was verified by sequencing. All of the primers used are listed in Table 1. Finally, the plasmids were transformed into *Escherichia coli* Rosetta pLys-S cells (Novagen) to express the protein.

After induction with 1 mM IPTG, the culture (1 l) was grown at 30 °C for 5 h. The bacterial cells were recovered by centrifugation (in a JLA 81000 rotor, Beckman-Coulter, at 6 Krpm for 15 min) and disrupted by sonication (VibraCell 75042 sonicator, Bioblock Scientific, tip model CV33). The sample was centrifuged in a JA 25–50 rotor (Beckman-Coulter), at 20 Krpm for 30 min. The supernatant was filtered using 0.45 µm syringe-driven filters (Jet Biofil) and loaded in a His-Trap column (GE Healthcare) equilibrated with 350 mM NaCl, 50 mM Tris pH 7.5, 1 mM TCEP and 10 mM Imidazole. The proteins were eluted with 350 mM NaCl, 50 mM Tris pH 7.5, 1 mM TCEP and 150 mM Imidazole. After concentration in an Amicon Ultracel 10,000 MCWO concentrator (Millipore), the sample was loaded into a Superdex 75 16/60 column (GE Healthcare), equilibrated with 150 mM NaCl, 20 mM Tris pH 7.5 and 1 mM TCEP. The protein was eluted in a single peak. Finally, the pooled peak fractions were concentrated to 40 mg/ml, as previously described. The purification process was carried out at 4 °C, and the purity was determined by SDS-PAGE (Fig. 1).

### Determination of the muralytic activity of endolysins ElyA1 and ElyA2

Muralytic activity was determined using the Gram-negative overlay method described by Schmitz *et al*.^54^. Briefly, two clinical isolates of *A. baumannii*, MAR001 and PAU002, were grown to stationary phase (10^9^ CFU/ml) in LB, pelleted and resuspended in PBS buffer pH 7.4. Agar was added directly to the bacterial suspension at a concentration of 0.8%, and the mixture was autoclaved for 15 min at 120 °C. The medium containing the disorganized cells and the exposed peptidoglycan was solidified in Petri dishes, and aliquots (50 μg) of endolysin or the endolysin buffer (as a negative control) were spotted on the surface.

The muralytic activity was measured using as target a culture of *A. baumannii* Ab105 treated with EDTA in order to permeabilize the outer membrane. An overnight culture of *A. baumannii* Ab105 was diluted 1:100 in LB medium and grown to exponential phase (0.3–0.4 OD600nm). The culture was centrifuged (3000 g, 10 min), and the resulting pellet was resuspended in 20 mM Tris-HCl buffer at pH 8.5 with 0.5 mM EDTA before being incubated for 30 min at room temperature. The pellet was recovered by centrifugation and washed twice in Tris-HCl buffer pH 8.5. Finally, the cells were resuspended in 20 mM Tris-HCl 150 mM NaCl pH8.5 and 25 µg/ml of endolysin ElyA1. The activity was measured by the turbidity reduction assay, as a decrease in the optical density measured at a wavelength of 600 nm (OD_600_) after incubation with shaking at 37 °C^17^. The OD_600_ was measured at intervals of 5 minutes for a period of 20 minutes and the time point of the highest activity was established. The optimal pH and temperature for endolysin activity were determined in the turbidity reduction assay. The reaction was carried out as previously described, with the Tris-HCl at different pH (range 6.5 to 9) and temperature (room temperature, 30 °C and 37 °C).

### Antibacterial assays

The antibacterial activity of the endolysin was assayed with all of the 67 clinical strains of *A. baumannii, P. aeruginosa* and *K. pneumoniae* (Table 1). The activity was determined using the turbidity reduction assay, as previously described, at pH 8.5 and 37 °C. The incubation times in the presence of EDTA varied according to the species assayed: 30 min for *A. baumannii* and *K. pneumoniae* and 15 min for *P. pneumoniae*.

### Broth microdilution checkerboard assay and microdilution test to determine minimum inhibitory concentrations (MICs)

This assay was conducted with the strains disaplying the highest and the lowest susceptibility to endolysin. All the strains tested were susceptible to colistin (Table S1), except three strains, which were colistin resistant: *A. baumannii* SOF004b, *P. aeruginosa* AUS034 and *K. pneumoniae* KP2. The effect of the interaction between endolysin and colistin was determined by the microdilution checkerboard assay. Seven serial double dilutions of endolysin and 6 of colistin were made with Mueller-Hinton Broth (MHB) in the wells of a 96-well microtiter plate. The wells were then inoculated with the test culture to a final concentration of 10^5^ colony forming units (cfu/ml). The MICs of colistin (0 to 2 µgml^−1^) and of the ElyA1 protein (3.125 to 200 µgml^−1^) were assayed independently in the same plate. The MIC was determined as the concentration of antimicrobial agent in the well in which no visible growth of bacteria was observed after incubation for 24 h at 35 °C.

### Time kill curve assay

Time kill curve assays were carried out with those strains in which the colistin MIC in the colistin-ElyA1 combinations was decreased by at least fourfold in the checkerboard assays. The assay was conducted according to previously described techniques^55^. Flasks of LB containing colistin and colistin plus endolysin at the concentration indicated in the checkerboard assay were inoculated with a 1:100 dilution of an overnight culture in stationary phase of the tested strain and incubated at 37 °C and 180 rpm in a shaking incubator. Aliquots were removed after 0, 6 and 24 h and were serially diluted and plated to produce colony forming units (cfu). Synergy was established when a ≤ 2 −log_10_ decrease in cells counts at 6 or 24 h in the antimicrobial combination relative to the most active single agent was observed. No effect was considered to have occurred when the counts were <2−log _10_ lower or higher relative to the culture with the single agent. Antagonism was defined when the counts in the culture with antimicrobial combination were ≥2−log _10_ higher than in the culture with single most active antimicrobial agent.

The reduction in the colistin MIC in combination with endolysins was also assayed by combining colistin with another endolysin, ElyA2, isolated from bacteriophage Ab105Φ2. The curve was constructed for the same strains and under the same conditions as for colistin + ElyA1.

### ***Galleria mellonella*infection model**

The *Galleria mellonella* model was an adapted version of that developed by Peleg *et al*.^56^ as well as in other studies with endolysins assays^57,58^. The procedure was as follows: twelve *G. mellonella* larvae, acquired from TRULARV (Biosystems Technology, Exeter, Devon, UK), were each injected with 10 μl of a suspension of *A. baumannii* GMA001, diluted in sterile phosphate buffer saline (PBS) containing 1 × 10^5^ CFU (±0. 5 log). The injection was performed with a Hamilton syringe (volume 100 μl) (Hamilton, Shanghai, China). One hour after infection, the larvae were injected with 10 μl of colistin (1/4 MIC) plus endolysin ElyA1 (25 µg/ml), colistin (1/4 MIC) plus endolysin ELyA2 (25 µg/ml), and colistin alone (as controls), all at the same concentrations used in the time kill curve. After being injected, the larvae were placed in Petri dishes and incubated in darkness at 37 °C. The number of dead larvae was recorded during 5 days. The larvae were considered dead when they showed no movement in response to touch^56^.

The mortality curves corresponding to the *in vivo Galleria mellonella* infection model were constructed using GraphPad Prism v.6, and the data were analysed using the Graham-Breslow-Wilcoxon test. In both cases, *p*-values < 0.05 were considered statistically significant, and the data were expressed as mean values.

### Mouse skin infection model

A superficial skin wound infection by tape stripping in mouse was done as previously described^59,60^, with some modifications. Female BALB/c mice (6–8 weeks old) were anaesthetized with an injection of ketamine (500 μg/mouse) and medetomidine (15 μg/mouse). Mice were shaved with an electric razor, and an area of skin of 2 cm^2^ was stripped with autoclave tape, until the skin was reddish and shiny. The tape stripped areas were cleaned with ethanol and allowed to dry. The area was then treated with 10 µl of a culture of *A. baumannii* GMA001 (1 × 10^8^ CFU/ml) or with PBS (in control mice). At 24 h post infection, the area was re-infected under the same conditions as before. The infection was established for another 24 h and the treatments were applied to groups of mice (n = 5); 3 groups were treated with colistin (1/4 MIC) in combination with 20 µl of endolysin ElyA1 (50 µg and 350 µg); a colistin control group was treated with 20 µl colistin alone (1/4 MIC); and a control group was treated with 20 µl of endolysin buffer. Three hours post-treatment the mice were euthanized with an overdose of thiopental sodium, and the skin in the wound area was excised and homogenized in sterile 0.9% NaCl, in a Retsch MM200 mixer mill. The homogenate was serially diluted and plated on agar MacConkey supplemented with ampicillin (50 µg/ml), in order to eliminate the normal skin flora, and to calculate the *A. baumannii* GMA001 CFUs in each skin sample.

### Mouse lung infection model

A culture of *A. baumannii* GMA001 was grown from a 1:100 dilution of an overnight culture to an OD_600_ of 0.7. The cultures were washed and suspended in PBS to obtain an inoculum of 4–6 × 10^7^ CFU in 40 µl per mouse.

Male BALB/c mice, 9–11 weeks old were anaesthetized by inhalation of sevofluorane (Zoetis, Madrid, Spain) and suspended by their incisors on a board in a semi-vertical position. The mice were infected by intratracheal instillation with 40 µl of a bacterial suspension (4–6 × 10^7^ CFUs). The mice were anaesthetized by inhalation of sevofluorane (Zoetis, Madrid, Spain) and divided into three groups (n = 10). At 3 h post-infection, the control group was treated by intranasal instillation of 30 µl endolysin buffer, the colistin control group was treated with colistin (1/4 MIC), and the treatment group was treated with a combination of colistin (1/4 MIC) and endolysin ElyA1 (350 µg). At this point, three mice were euthanized to determine the bacterial load in the lungs before treatment. Finally, 20 h after treatment, mice were euthanized with an overdose of sodium thiopental (Sandoz, Holzkirchen, Germany), and the lungs were extracted and homogenized in 1 ml of sterile 0.9% NaCl, in a Retsch MM200 mixer mill. The homogenate was serially diluted and plated on agar MacConkey for determination of the *A. baumannii* GMA001 CFUs in each lung sample.

All of the experiments with mice were conducted with the approval of and in accordance with the regulatory guidelines and standards established by the Animal Ethics Committee (INIBIC-CHUAC, Spain, project code 2016/R06).

## Supplementary information

Supplementary information.
