# Supplementary material for: In vitro and in vivo efficacy of combinations of colistin and different endolysins against clinical strains of multi-drug resistant pathogens
Source: Sci Rep. 2020 Apr 28;10:7163. doi: 10.1038/s41598-020-64145-7 (PMC7188820; doi:10.1038/s41598-020-64145-7)
Supplement: Supplementary file 1 — Supplementary information. [file 41598_2020_64145_MOESM1_ESM.docx]

| **Strains** |  | **Ceftazidime^a^** | **Imipenem^a^** | **Meropenem^a^** | **Tobramycin^a^** | **Amikacin^a^** | **Ciprofloxacin^a^** | **Colistin^a^** |
| --- | --- | --- | --- | --- | --- | --- | --- | --- |
| *A.baumannii* | GMA001 | >128 | 4 | 16 | <2 | <2 | >64 | <0.5 |
|  | PON001 | >128 | 8 | 8 | 8 | <2 | >64 | 1 |
| *P.aeruginosa* | AUS531 | 3 | 2 | 0.75 | 1 | 6 | 0.125 | 1 |
|  | AUS601 | >256 | >32 | >32 | 24 | >256 | 16 | 0.25 |
| *K.pneumoniae* | KP16 | >16 | >16 | >16 | >8 | >32 | >2 | 1 |
|  | KP17 | 1 | 16 | 8 | ≤1 | ≤4 | ≤0.06 | 1 |

**TABLE S1.  ^a^** MIC concentrations (mg/L)
